# Frontal cortex chitinase and pentraxin neuroinflammatory alterations during the progression of Alzheimer’s disease

**DOI:** 10.1186/s12974-020-1723-x

**Published:** 2020-02-17

**Authors:** Marta Moreno-Rodriguez, Sylvia E. Perez, Muhammad Nadeem, Michael Malek-Ahmadi, Elliott J. Mufson

**Affiliations:** 1 Department of Neurobiology and Neurology, Barrow Neurological Institute, St. Joseph’s Hospital and Medical Center, 350 W. Thomas Rd., Phoenix, AZ 85013 USA; 2 Banner Alzheimer’s Institute, Phoenix, AZ USA

**Keywords:** Alzheimer’s disease, Astrocytes, Chitinase, Cognitive impairment, Frontal cortex, Gray matter, Microglia, Neuroinflammation, Pentraxin, White matter

## Abstract

**Background:**

Chitinase 3-like 1 (CHI3L1), chitinase 3-like 2 (CHI3L2), and neuronal pentraxin II (NPTX2) are inflammatory biomarkers of Alzheimer’s disease (AD). Although studies have demonstrated that cerebrospinal fluid levels of these proteins are changed in AD, no studies have undertaken a detailed examination of alterations in protein levels, cellular expression, and interaction with amyloid in the brain during the progression of AD.

**Methods:**

The study evaluated levels of both CHI3L1 and CHI3L2, NPTX2, ionized calcium-binding adapter molecule 1 (Iba1), complement component 1q (C1q), glial fibrillary acidic protein (GFAP), and CD44, in the frontal cortex of people who died with an antemortem clinical diagnosis of no cognitive impairment (NCI), mild cognitive impairment (MCI), mild/moderate AD (mAD), and severe AD (sAD) using immunoblot and immunohistochemical techniques.

**Results:**

CHI3L1-immunoreactive (-ir) astrocyte numbers were increased in the frontal cortex and white matter in sAD compared to NCI. On the other hand, increases in GFAP and Iba1-ir cell numbers were observed in MCI compared to NCI but only in white matter. Western blot analyses revealed significantly lower frontal cortex CHI3L2 levels, whereas CD44 levels were increased in sAD. No significant differences for CHI3L1, GFAP, C1q, and NPTX2 protein levels were detected between clinical groups. Strong significant correlations were found between frontal cortex CHI3L1 and Iba1-ir cell numbers in white matter and CHI3L1 and C1q protein levels in the early stages of the disease. C1q and Iba1, CD44 with CHI3L2, and GFAP protein levels were associated during disease progression. CHI3L1 and Iba1 cell numbers in white matter showed a significant associations with episodic memory and perceptual speed.

**Conclusions:**

White matter CHI3L1 inflammatory response is associated with cognitive impairment early in the onset of AD.

## Introduction

Alzheimer’s disease (AD) is an irreversible, progressive neurodegenerative disorder resulting in cognitive decline leading to extreme societal costs [1, 2]. With a rapidly growing older population worldwide, defining the cellular mechanism(s) driving the onset of AD is of paramount importance. AD-related cognitive decline is manifested by deficits in working memory and executive function modalities associated, in part, with the frontal cortex (FC) [3]. The FC is a hub of the default mode network that displays extensive amyloid pathology associated with cognitive decline early in AD [4, 5]. However, the underlying cellular mechanism(s) driving FC dysfunction remains unknown.

AD is pathologically characterized by the accumulation of amyloid-beta (Aβ) plaques, tau neurofibrillary tangles (NFTs), synaptic and neuronal loss, glial activation, and neuroinflammation [6–11]. Aβ plaques and NFTs induce an immune response associated with astroglia and microglia activation and increased inflammatory mediators, tumor necrosis factor α [12], S100, interleukin 1 [13, 14], such as triggering receptor expressed on myeloid cells 2 (TREM2) [15, 16], and complement activation (C1q to C5b-9) [17] in AD [18–20]. Recently, the chitinase family of inflammatory proteins, particularly chitinase 3-like 1 (CHI3L1, YKL-40, or HC gp-39), chitinase 3-like 2 (CHI3L2 or YKL-39), and pentraxin II (NPTX2 or Narp), a member of the pentraxin family, has been associated with AD pathogenesis [21, 22]. Although their functions are not well understood, it is hypothesized that chitinases are involved in pro-inflammatory and pro-angiogenic tissue remodeling in cancer [23, 24] as well as in several neurodegenerative diseases [25–32]. CHI3L1 found in the cerebral spinal fluid (CSF) obtained from preclinical and prodromal cases of AD [33–35] has been suggested as a biomarker for discerning cognitively normal from mild cognitive impairment (MCI) individuals [25, 36, 37]. Human tissue-based studies revealed that CHI3L1 is expressed in astrocytes in close apposition to blood vessels, Aβ plaques and NFTs in AD [38]. However, the role that chitinases play in cognitive impairment during the progression of AD remains under-investigated.

The protein NPTX2, a member of the pentraxin family [39, 40], is involved in excitatory synapse formation [41, 42] and has been implicated in the regulation of neuroinflammatory responses associated with trauma and neurological disease. For example, the deletion of NPTX2 produces an alteration of microglial activation following sciatic nerve transection [43]. Interestingly, reduced CSF levels of NPTX2 were associated with medial temporal lobe atrophy and cognitive decline in AD [44].

Although CSF chitinase and NPTX2 neuroinflammatory proteins are putative biomarkers related to the pathogenesis and cognitive decline seen in AD [45, 46], there are virtually no detailed clinicopathological studies of these proteins in brain tissue during the clinical onset of AD. Therefore, we examined CHI3L1 and NPTX2 protein levels and cellular expression in the FC, a region affected early by plaque pathology during the progression of AD. These proteins were compared with other glial neuroinflammatory markers such as microglial ionized calcium-binding adapter molecule 1 (Iba1), complement component 1q (C1q), TREM2, astrocytic glial fibrillary acidic protein (GFAP), and glial surface adhesion glycoprotein CD44, which interacts directly with CHI3L1 [47] and drives immune responses in the central nervous system [48]. These proteins were quantified using immunoblotting and immunohistochemistry or immunofluorescence techniques. Changes in these markers were correlated with case demographics and cognitive and neuropathological variables.

## Methods

### Subjects

The individuals used in this study were selected based upon a premortem clinical diagnosis of no cognitive impairment (NCI, *n* = 15), mild cognitive impairment (MCI, *n* = 15), and mild to moderate AD (mAD, *n* = 14) from the Rush Religious Orders Study (RROS) cohort (Table 1) and severe AD (sAD, *n* = 12) from the Rush Alzheimer’s Disease Center. Subject selection was not based upon NFT Braak, amyloid Thal, or ApoE criteria. Association of these variables with the present findings was evaluated after the study was completed to avoid pathological bias. The Human Research Committees of Rush University Medical Center approved this study, and informed consent for research and autopsy was obtained from RROS participants or family/guardians.

**Table 1:** Demographic, cognitive, and neuropathological characteristics

		NCI (*N* = 15)	MCI (*N* = 15)	mAD (*N* = 14)	Total (*N* = 44)	Overall *p* value	Pairwise comparisons
Age (years) at death	Mean ± SD Range	85.20 ± 4.27 (78–94)	86.65 ± 5.02 (79–95)	88.29 ± 4.70 (76–95)	86.68 ± 4.74 (76–95)	0.16*	–
Number of males (%)		6 (50)	6 (40)	5 (36)	17 (39)	0.96†	–
Years of education	Mean ± SD Range	17.20 ± 3.80 (10–23)	17.53 ± 2.97 (10–20)	18.57 ± 3.98 (9–26)	17.75 ± 3.56 (9–26)	0.67*	–
Number ApoE ε4 allele (%)		0 (0)	4 (27)	4 (27)	8 (18)	0.08†	–
MMSE	Mean ± SD Range	28.13 ± 1.51 (26–30)	27.13 ± 2.67 (22–30)	21.29 ± 4.83 (14–28)	25.61 ± 4.38 (14–30)	< 0.001*	NCI, MCI > mAD
GCS	Mean ± SD Range	− 0.02 ± 0.27 (− 0.36–0.42)	− 0.45 ± 0.35 (− 1.20–0.04)	− 1.15 ± 0.53 (− 2.07–0.08)	− 0.52 ± 0.60 (− 2.07–0.42)	< 0.001*	NCI > MCI > mAD
Episodic memory *z*-score	Mean ± SD Range	0.39 ± 0.37 (− 0.41–0.91)	− 0.27 ± 0.43 (− 1.00–0.64)	− 1.47 ± 0.86 (− 3.11–0.08)	− 0.43 ± 0.96 (− 3.11–0.91)	< 0.001*	NCI > mAD
Semantic memory *z*-score	Mean ± SD Range	− 0.28 ± 0.76 (− 1.44–1.08)	− 0.41 ± 0.60 (− 1.58–0.60)	− 0.72 ± 0.83 (− 2.96–0.16)	− 0.47 ± 0.74 (− 2.96–1.08)	0.30*	–
Working memory *z*-score	Mean ± SD Range	− 0.09 ± 0.47 (− 0.96–0.48)	− 0.35 ± 0.69 (− 1.32–1.08)	− 0.60 ± 0.62 (− 1.78–0.57)	− 0.34 ± 0.62 (− 1.78–1.08)	0.10*	–
Perceptual speed *z*-score	Mean ± SD Range	− 0.56 ± 0.65 (− 1.63–0.45)	− 0.90 ± 0.60 (− 2.08–0.04)	− 2.06 ± 0.88 (− 3.38 − − 0.54)	− 1.15 ± 0.95 (− 3.38–0.45)	< 0.001*	NCI, MCI > mAD
Visuospatial *z*-score	Mean ± SD Range	− 0.32 ± 0.59 (− 1.24–0.74)	− 0.84 ± 0.63 (− 1.68–0.44)	− 0.97 ± 0.64 (− 2.12–0.30)	− 0.70 ± 0.67 (− 2.12–0.74)	0.03*	NCI > MCI, mAD
Post-mortem interval (h)	Mean ± SD Range	5.55 ± 2.16 (1–9)	5.74 ± 2.18 (2–10)	4.69 ± 2.40 (1–10)	5.35 ± 2.24 (1–10)	0.27*	–
Brain weight (g)	Mean ± SD Range	1190.80 ± 89.54 (1000–1320)	1179.47 ± 142.41 (990–1480)	1144.79 ± 112.51 (962–1320)	1172.30 ± 115.86 (962–1480)	0.54*	–
Distribution Braak scores	0	0	0	0	0	0.74†	–
	I/II	2	2	4	8		
	III/IV	12	11	7	30		
	V/VI	1	2	3	6		
NIA Reagan	No AD	0	0	0	0	0.30†	–
	Low	7	8	4	19		
	Intermediate	8	5	7	20		
	High	0	2	3	5		
CERAD	No AD	4	6	2	12	0.32†	–
	Possible	6	6	5	17		
	Probable	3	1	1	5		
	Definite	2	2	6	10		

*Kruskal-Wallis test with Conover-Inman test for pairwise comparisons; ^†^chi-square *p* value; one AD individual did not have APOE genotyping; severe AD cases not shown in table

### Clinical and neuropathological characteristics

Table 1 shows the demographic, clinical and neuropathological characteristics of the RROS cases examined. Clinical and neuropathological criteria for NCI, MCI, and AD diagnosis were reported previously [49–53]. Briefly, after a review of the clinical data and examination of the participant, clinical diagnoses were made by a board-certified neurologist with expertise in gerontology. The neurologist reviewed medical history, medication use, neurologic examination information, results of cognitive performance testing, and the neuropsychologist’s opinion of cognitive impairment and the presence of dementia. Each participant was evaluated in his/her home, emphasizing clinically relevant findings. AD diagnosis of dementia followed the recommendations of the joint working group of the National Institute of Neurological and Communicative Disorders and the Stroke and the Alzheimer’s Disease and Related Disorders Association (NINCDS/ADRDA) [54]. Although there are no consensus criteria for the clinical classification of mild cognitive impairment, the criteria used in the present study are compatible with those used by many others in the field to describe those persons who are not cognitively normal, but do not meet the accepted criteria for dementia [55–60]. Here, MCI was defined as persons rated as impaired on neuropsychological testing by the neuropsychologist but were not found to have dementia by the examining neurologist. Average time from the last clinical evaluation to death was ~ 8 months.

Clinical neuropsychological testing included Mini-Mental State Examination, global cognitive score, composite z-score compiled from 19 cognitive tests [61], and z-scores from episodic memory, semantic memory, working memory, perceptual speed, and visuospatial tests. Postmortem neuropathology was performed as reported previously [15, 49–51, 53], which included Braak staging [62], NIA-Reagan criteria [63], and the Consortium to Establish a Registry for Alzheimer’s Disease (CERAD) [64]. A board-certified neuropathologist excluded cases with other pathologies (e.g., cerebral amyloid angiopathy, vascular dementia, dementia with Lewy bodies, hippocampal sclerosis, Parkinson’s disease, and large strokes) and those treated with acetylcholinesterase inhibitors. The RROS neuropathology core includes the transactive response DNA-binding protein of 43 kDa (TDP-43) [65] in their diagnosis. A similar detailed clinical and neuropathological evaluation were not available for the sAD cases.

### Quantification of pathological lesions

Neuritic plaques, diffuse plaques, and NFT counts were performed within a 1-mm^2^ area (×100 magnification) per neocortical region blinded to clinical data [66–68] using Bielschowsky silver stain and AT8 immunohistochemistry. Amyloid precursor protein (APP)/Aβ was used to analyze the Aβ load [69]. Standardized plaque and tangle counts from each area were converted to standard scores by dividing the standard deviation of mean raw counts per marker and region from the entire deceased cohort. Quantitation of amyloid load was performed by using images from Aβ labeled sections [69] to determine percent area occupied using Object-Image 1.62p15 [70].

### Antibodies

Characteristics of primary and secondary antibodies used for immunohistochemical, immunofluorescence, and western blotting experiments are described in Table 2.

**Table 2:**
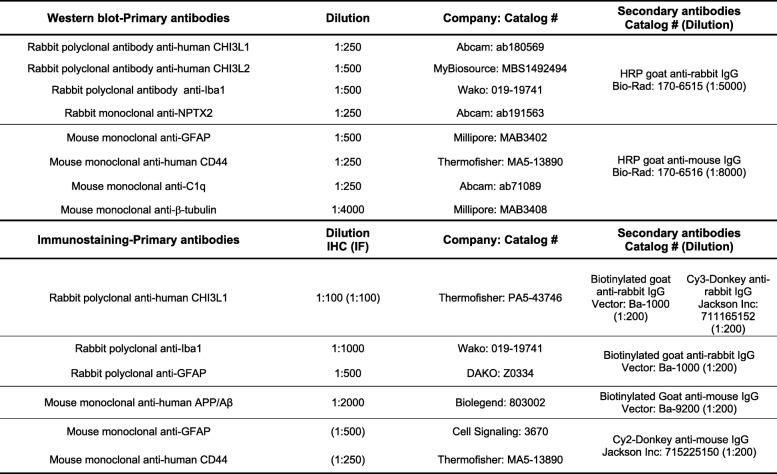
Antibodies

### Western blotting

FC (Brodmann’s area 10) CHI3L1, CHI3L2, NPTX2, GFAP, C1q, Iba1, and CD44 protein levels were measured in 15 NCI, 15 MCI, 13 mAD, and 7 sAD samples [15]. Briefly, frozen samples were homogenized (150 mg/mL) in phosphate buffer containing protease inhibitors (Sigma, St. Louis, MO) and denatured in SDS loading buffer to a final concentration of 5 mg/ml. Proteins (50 μg/sample) were separated by SDS-PAGE (Lonza, Rockland, ME) and electrophoretically transferred to polyvinylidene fluoride membranes (Millipore) [71]. Membranes were blocked in tris-buffered saline/0.05% Tween-20/5% milk (1 h) at room temperature (RT). Antibodies were added to blocking buffer and membranes incubated overnight (4 °C), washed, incubated with horseradish peroxidase-conjugated goat anti-mouse IgG secondary antibody or goat anti-rabbit IgG secondary antibody at room temperature (RT), visualized by chemiluminescence (Kodak Image Station 440CF; Perkin-Elmer, Wellesley, MA), and quantified with Kodak 1. Protein signals were quantified and normalized to β-tubulin across groups in three independent experiments [71]. Controls consisted of either pre-absorption or deletion of the primary antibody.

### Immunohistochemistry

Two 8-μm-thick paraffin-embedded FC sections were processed for immunocytochemistry to visualize inflammatory markers, NFTs, and plaques. We chose CHI3L1 as opposed to CHI3L2 for immunohistochemical staining since the commercial CHI3L2 antibodies failed to react with the human brain tissue. Briefly, sections were pretreated with either citric acid (pH = 6) for 20 min as antigen retrieval for the CHI3L1 antibody and 80% formic acid for the APP/Aβ (6E10) antibody. Afterward, sections were incubated with primary antibodies against rabbit anti-CHI3L1, rabbit anti-GFAP, rabbit anti-Iba1, and mouse anti-APP/Aβ or AT8 overnight at RT in a tris-buffered saline/0.25% Triton X-100/1% goat serum solution. After several washes in tris-buffered saline, tissue samples were incubated with a goat anti-rabbit/anti-mouse biotinylated secondary antibody, then incubated in Vectastain ABC kit (1 h) (Vector Labs) and developed in acetate-imidazole buffer containing 0.05% 3,3′-diaminobenzidine tetrahydrochloride (DAB, Sigma, MO).

### Dual immunostaining

After visualization of APP/Aβ (see above), the tissue was incubated with an avidin/biotin blocking kit (Vector Labs) and second primary antibody (rabbit anti-CHI3L1) overnight at RT [72]. The next day tissue was placed in the appropriate biotinylated secondary antibody for 1 h at RT, incubated in ABC kit solution, and developed in acetate-imidazole buffer containing 0.05% 3,3′-DAB and 1% of nickel ammonium sulfate (Sigma). Dual staining produced a two-colored profile: APP/Aβ (brown) and CHI3L1 (black). Immunohistochemical controls consisted of primary antibody omission resulting in the absence of immunoreactivity. Additional sections were stained with Gill’s hematoxylin (1 min) to identify cortical layers.

### Immunofluorescence

Sections were double-labeled with a mouse anti-GFAP or anti-CD44 and a rabbit anti-CHI3L1 antibody overnight. The appropriate secondary antibody was applied [Cy3-donkey anti-rabbit IgG for CHI3L1 and Cy2-donkey anti-mouse IgM for GFAP] and incubated in a 0.1 thioflavin solution (10 min) to visualize aggregated amyloid. Auto-fluorescence was blocked with Auto-fluorescence Eliminator Reagent (Millipore) and sections cover-slipped with aqueous mounting media (Thermo Scientific). Dual immunofluorescence was visualized, and images were acquired using the Revolve Fluorescent Microscope (Echo Laboratories, San Diego, CA, USA) with excitation filters 405, 489, and 555 nm for thioflavin, Cy2, and Cy3, respectively.

### Quantitation of CHI3L1, GFAP, and Iba1 profiles

CHI3L1 and GFAP-immunoreactive (-ir) cell counts were performed in five random fields within an area of 0.14 mm^2^ per field in both gray matter and white matter (WM) in two sections from the same 15 NCI, and MCI, 14 mAD, and 5 sAD cases. APP/Aβ plaque loads and numbers and counts of AT8-positive cells within the gray matter were performed as above using tissue from the same cases. Counts of Iba1 profiles in gray matter and WM were evaluated in 6 cases/per clinical group. FC gray matter CHI3L1-ir cell counts were performed in the different cortical layers (I–II, III–IV, V–VI, and WM), while GFAP and Iba1 counts, were counted independent of cortical layer. All images and counts were acquired using a Nikon Eclipse 80i coupled with NIS-Elements Imaging software (Nikon Americas Inc., NY). Images were corrected for contrast and luminosity using Adobe Photoshop CS4 software (Adobe Systems Inc., CA).

### Statistical analysis

Data evaluated across clinical groups used Mann-Whitney, Kruskal-Wallis, Chi-square, and Wilcoxon signed-rank test followed by Conover-Inman, Holm-Šidák, Tukey, and Dunn’s post hoc tests for multiple comparisons and Spearman rank for correlations (Sigma Plot 12.5, Systat Software, San Jose, CA, USA). A false discovery rate was used to adjust for multiple comparisons between correlations. Statistical significance was set at *p* < 0.05 (two-tailed) and data graphically represented using GraphPad Prism 5 (GraphPad Software, San Diego, CA, USA).

## Results

### Demographic, cognitive, and neuropathological characteristics

RROS clinical groups did not differ by age, gender, education, postmortem interval, or brain weight. There were no significant differences in the number of cases carrying the ApoE ε4 allele. Mini-Mental State Examination score, global cognitive score, and perceptual speed were significantly lower (*p* < 0.001) in mAD compared to MCI and NCI cases. Episodic memory z-score was significantly lower (*p* < 0.001) in mAD compared t NCI, and the visuospatial z-score was significantly lower (*p* < 0.03) in mAD and MCI compared to that of NCI. Braak scores, CERAD, and NIA Reagan diagnosis did not differ among the clinical groups. Neuropathology revealed that 80% of NCI, 73.3% of MCI, and 50% of mAD cases were Braak stages III/IV. Using NIA-Reagan criteria, 100% of NCI, 86% of MCI, and 78.5% of mAD were classified as low to intermediate likelihood of AD. CERAD criteria revealed that 33.3% of NCI, 20% of MCI, and 50% of mAD cases were probable or definitive AD (Table 1).

The sAD cases averaged 78.33 ± 4.47 years of age at death (range 71–86 years), 5.55 ± 3.34 h for postmortem interval (range 2–12 h), 1122.72 ± 124 g (range 1320–980 g) brain weight, 58.3% were female and average MMSE was 2.2 (range 0–9).

### FC amyloid plaque and NFT pathology

FC diffuse and neuritic plaques were observed in 69.2% of NCI, 53.3% of MCI, and 100% of mAD RROS cases, while only 23% of NCI, 35.71% of MCI, and 45.5% of mAD displayed NFTs. All severe AD cases displayed neuritic plaques and NFTs.

### FC CHI3L1, GFAP, and Iba1 distribution and counts during AD progression

**Fig. 1:**
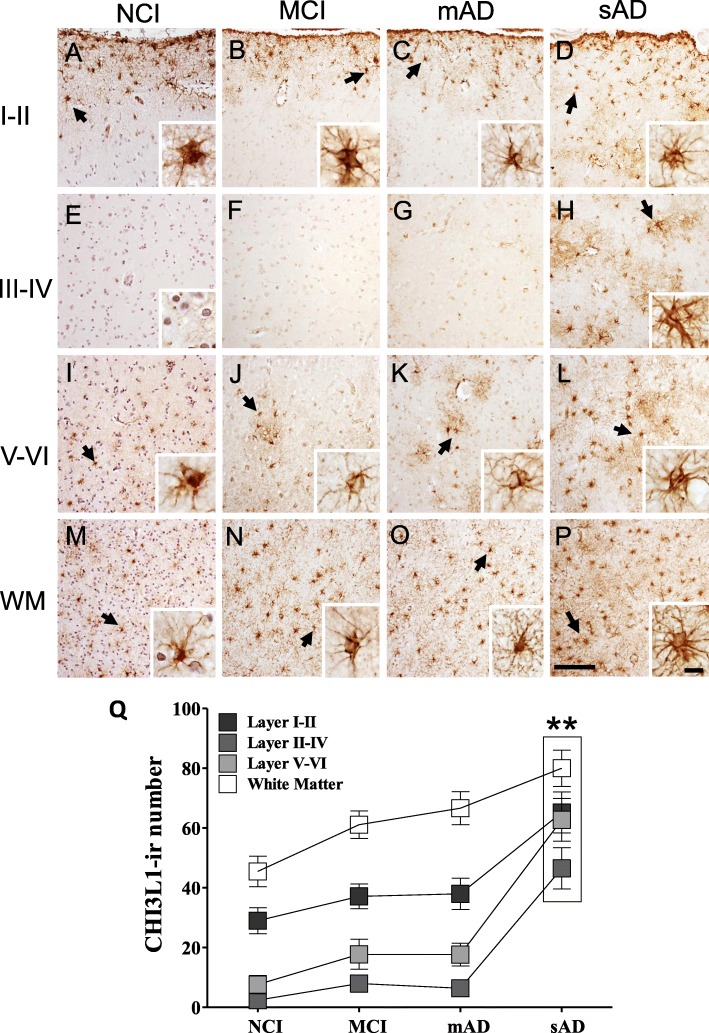
**a**–**h** Photomicrographs showing immunolabeling for CHI3L1 in the frontal cortex (FC) layers I–II, III–IV, and V–VI as well as WM in NCI, MCI, mAD, and sAD cases. Note CHI3L-ir cells appear mainly in layer I–II and WM in NCI, MCI, and mAD, while in sAD cells were also observed in layers III–IV and V–VI. Insets show high-power images of CHI3L-ir cells (arrows) in panels **a**–**d**, **h**, and **i**–**p**. **q** Graphic representation displaying the average numbers of CHI3L1-ir cells in the FC layers and WM in NCI, MCI, mAD, and sAD. Note the significantly higher numbers of CHI3L1-ir-positive cells in all FC layers and WM in sAD compared to NCI (*p* = 0.003). The tissue in **a**, **e**, **i**, and **m** panels were counterstained with Gill’s hematoxylin. Scale bar in panel **p** = 100 μm and inset = 10 μm, which applies to all other panels and insets in the figure. Abbreviations: NCI, no cognitive impairment; MCI, mild cognitive impairment; mAD, mild to moderate AD; sAD, severe AD

**Fig. 2:**
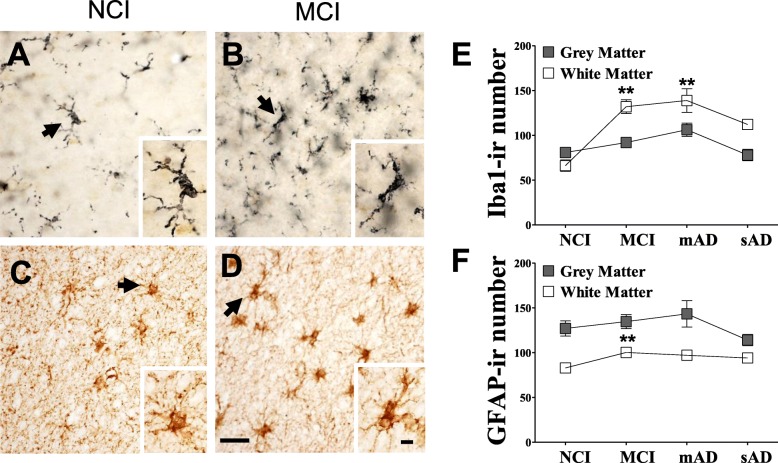
Photomicrographs showing Iba1 immunoreactive (-ir) microglia (**a**, **b**) and GFAP-ir astrocytes (**c**, **d**) in FC WM in NCI (**a**, **c**) and MCI (**b**, **d**) cases. Note that many more Iba1-ir microglia and GFAP-ir astrocytes were observed in MCI than NCI. Insets show high-power images of Iba1-ir microglia (**a**, **b**) and GFAP-ir astrocytes (arrows) (**c**, **d**). **e**, **f** Graphic representations showing the average number of Iba1-ir microglia (**e**) and GFAP-ir astrocytes (**f**) in WM across clinical groups. The iba1-ir numbers were significantly higher in MCI and mAD compared to NCI (*p* < 0.01), whereas the GFAP-ir cell numbers were significantly higher in MCI compared to NCI (*p* = 0.003). Scale bars in panel **d** = 25 μm and 5 μm in inset **d**, which apply to all panels and insets in this figure. Abbreviations: NCI, non-cognitive impairment; MCI, mild cognitive impairment

The topographic location of CHI3L1-ir cells was examined in FC white and gray matter across groups. In NCI (Fig. 1a, e, i, m), MCI (Fig. 1b, f, j, n) and mAD (Fig. 1c, g, k, o) numerous CHI3L1-ir astrocytes were observed in layers I and II and WM with lesser numbers in layers III–VI. Statistical analysis revealed that the number of CHI3L1-ir cells were significantly greater in all lamina and WM in sAD (Fig. 1d, h, l, p) compared to NCI (*p* < 0.01). In contrast, the numbers of Iba1-positive (Fig. 2a, b, e, *p* < 0.01) and GFAP-positive glial cells (Fig. 2c, d, f, *p* < 0.01) were significantly increased in MCI WM compared to that in NCI. In addition, WM Iba1-ir cells were significantly higher in mAD compared to NCI (Fig. 2e, *p* < 0.01).

**Fig. 3:**
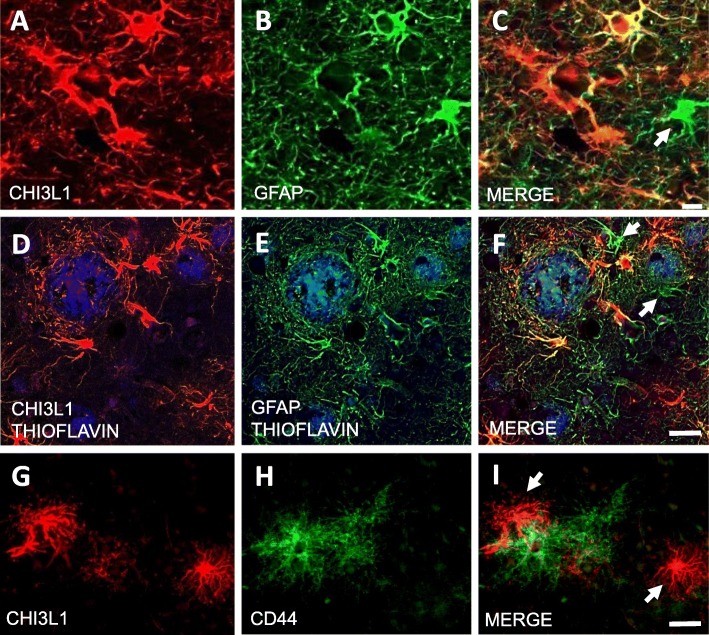
**a**–**i** Double immunofluorescent images show single CHI3L1 (**a**, **d**, **g**) (red), GFAP (**b**, **e**) (green), and CD44 (**h**) (green) labeling and merged (**c**, **f**, **i**) (orange) images in the FC in sAD. Plaques were stained with thioflavin (blue). Note that not all GFAP-positive astrocytes (white arrows) were CHI3L1-positive (**g**–**i**), and not all the CHI3L1-positive astrocytes (white arrow) were CD44-positive. Scale bar in **c** = 10 μm and applies to panels **a** and **b**, and 30μm scale bars in **f** and **i** also apply to **d**, **e**, **g**, and **h**

Dual immunofluorescence revealed that not all GFAP-ir astrocytes associated with plaques were also CHI3L1-positive (Fig. 3a–f), indicating that CHI3L1 co-occurs within a subset of GFAP-ir astrocytes regardless of amyloid plaques. Although a similar cortical distribution of CHI3L1-ir cells and the adhesion surface astrocyte marker CD44 was seen, not all CHI3L1-ir astrocytes co-expressed CD44 across groups (Fig. 3g–i).

**Fig. 4:**
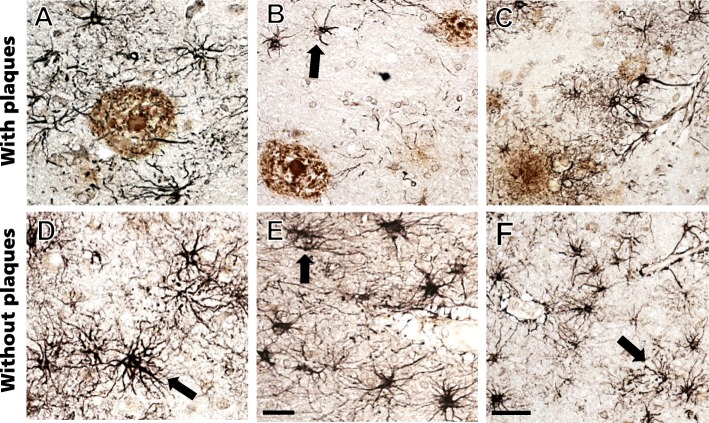
**a**–**f** Photomicrographs of astrocytes dual immunolabeled for CHI3L1 (blue/black) and APP/Aβ (brown) seen in plaques (**a**–**c**) as well as in areas without plaques (**d**–**f**) in sAD. Note that not all CHI3L1-positive astrocytes were associated with amyloid-positive plaques and blood vessels (arrows). Scale bar in **e** = 50 μm and applies to **a**, **b**, **d**. Scale bar in **f** = 100 μm and applies to **c**

Although not all CHI3L1-ir astrocytes were associated with amyloid plaques (Fig. 4a–c), CHI3L1-ir astrocytes in gray and WM were associated with blood vessels (Fig. 4d–f).

### FC neuroinflammatory protein levels during the progression of AD

**Fig. 5:**
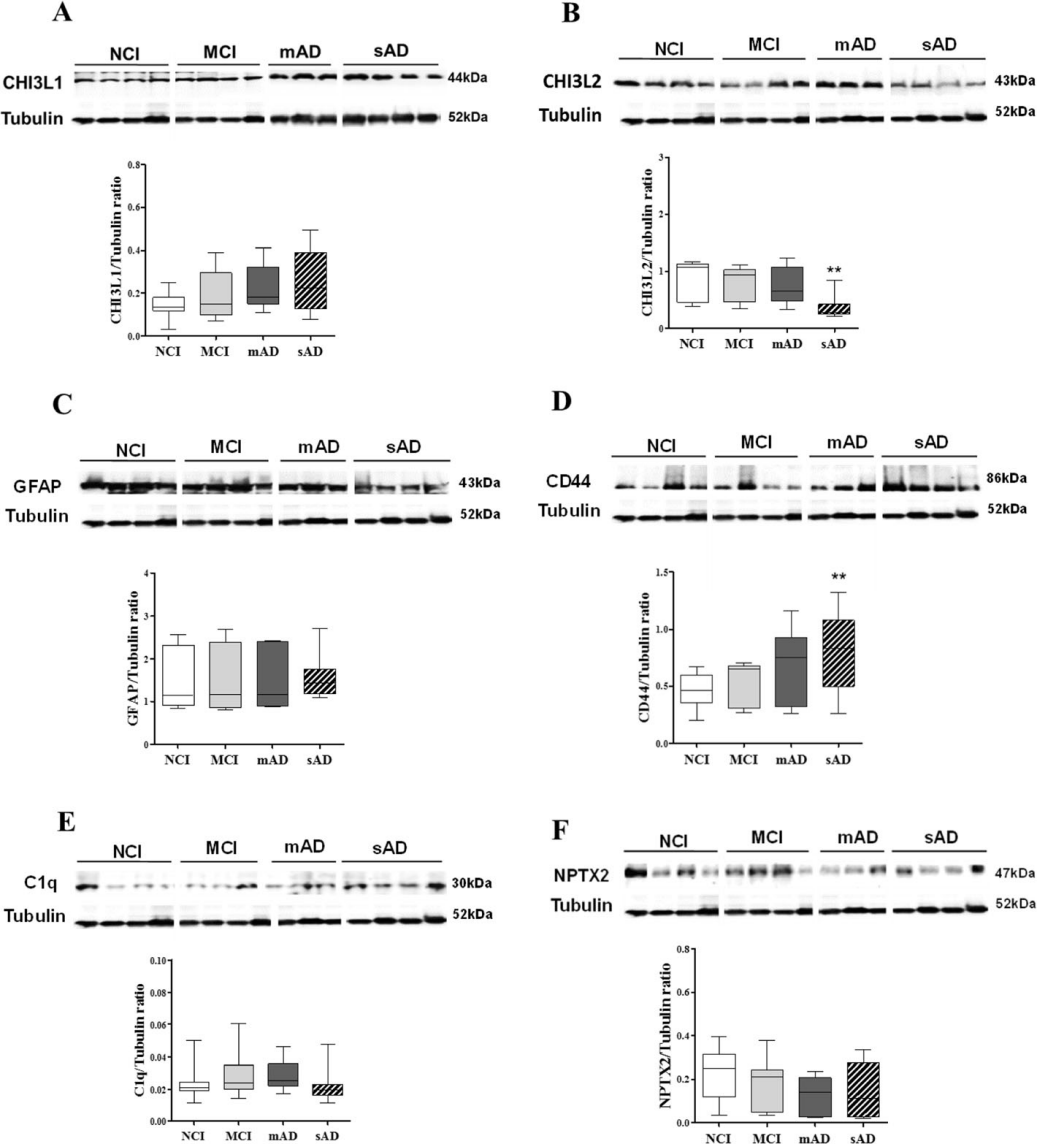
Representative immunoblots and box plots showing FC levels of CHI3L1 (**a**), CHI3L2 (**b**), GFAP (**c**), CD44 (**d**), C1q (**e**), and NPTX2 (**f**) in NCI, MCI, mAD, and sAD. β-tubulin was used to normalize the immunoreactive signal obtained by densitometry. Statistical analysis revealed that CHI3L1, NPTX2, GFAP, and C1q protein levels were stable across clinical groups, whereas there was a significant decrease in CHI3L2 levels, and CD44 significantly increased in sAD compared to NCI (*p* = 0.001). Abbreviations: NCI, non-cognitive impairment; MCI, mild cognitive impairment; mAD, mild to moderate AD; sAD, severe AD

Although western blot analysis revealed no significant differences in FC CHI3L1 levels between groups, there was a trend towards an increase in AD (Fig. 5a). Conversely, CHI3L2 protein levels were significantly reduced between NCI and sAD (Fig. 5b, *p* < 0.01). GFAP levels were unchanged across groups (Fig. 5c), whereas CD44 protein levels were significantly increased in sAD than in NCI (Fig. 5d, *p* < 0.01). C1q and NPTX2 did not change across clinical groups (Fig. 5e, f). A subanalysis comparing protein levels between low (I–III) and high (IV–VI) Braak scores within each clinical group [73] found that NPTX2 levels were significantly higher in mAD cases with high rather than low Braak scores (*p* = 0.001).

### Neuroinflammatory, clinical, and neuropathological associations

**Fig. 6:**
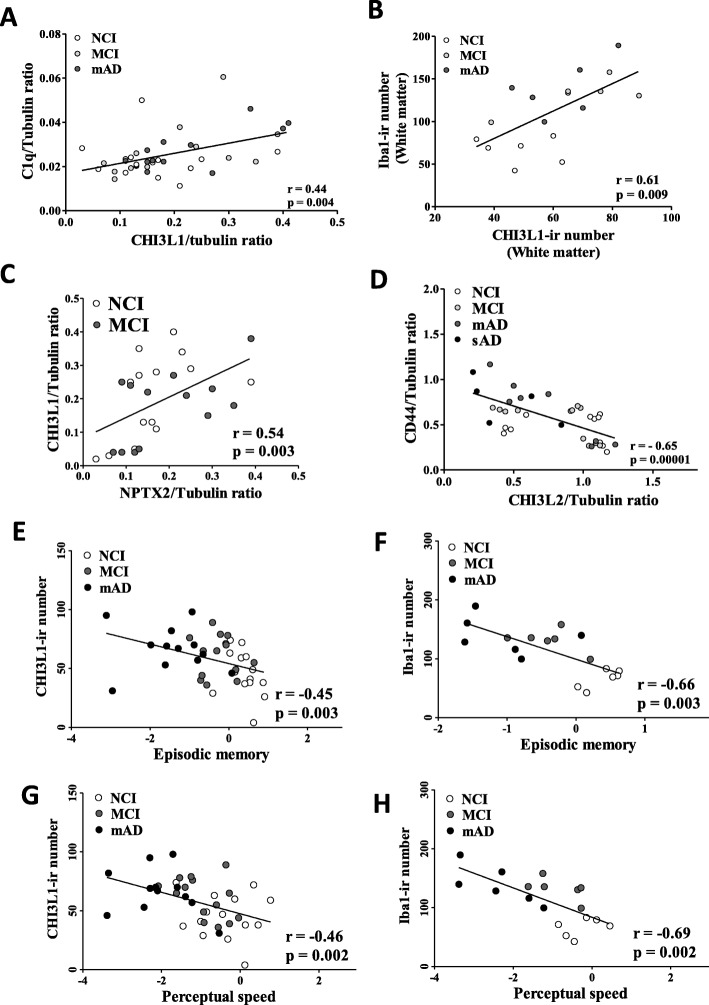
**a**, **b** Linear regression shows a significant correlation between CHI3L1 and C1q protein levels (**a**), Iba1-ir and CHI3L1-ir cell numbers (**b**) across NCI, MCI, and mAD. **c** A significant positive correlation was observed between CHI3L1 and NPTX2 (*r* = 0.054, *p* = 0.0003) protein levels across NCI and MCI, whereas a negative correlation was seen between CD44 and CHI3L2 (**d**, *r* = − 0.65, *p* < 0.0001) protein levels across the four clinical groups. In addition, positive correlations were observed between CHI3L1-ir (**e**, **g**) and Iba1-ir microglia cell numbers (**f**, **h**) with episodic memory (*p* = 0.003; **e**, **f**) and perceptual speed (*p* = 0.002; **g**, **h**) across NCI, MCI, and mAD. Abbreviations: NCI, noncognitive impairment; MCI, mild cognitive impairment; mAD, mild to moderate AD; sAD, severe AD

A significant correlation was found between CHI3L1 and C1q protein levels (Fig. 6a, *r* = 0.44, *p* = 0.004) and CHI3L1- and Iba1-ir cell numbers in WM (Fig. 6b, *r* = 0.61, *p* = 0.009) across NCI, MCI, and mAD. We found a significant correlation between NPTX2 and CHI3L1 protein levels across NCI and MCI (Fig. 6c, *r* = 0.54, *p* = 0.003), but not mAD. C1q and Iba1 (*r* = 0.57, *p* = 0.00005), CHI3L2 and CD44 (Fig. 6d, *r* = − 0.65, *p* = 0.00001), and GFAP and CD44 protein levels (*r* = − 0.49, *p* = 0.002) correlated across groups. Total numbers of amyloid plaque and NFTs did not correlate with CHI3L1, CHI3L2, GFAP, and C1q protein levels at any clinical stage. By contrast, we found a significant correlation between NPTX2 and total plaque load (*r* = − 0.46, *p* = 0.01) and diffuse plaque number (*r* = − 0.50, *p* = 0.007). In addition, a strong association was found between CHI3L2 and age (*r* = − 0.44, *p* = 0.0048) and CHI3L1- and Iba1-ir cell numbers were significantly correlated with episodic memory (CHI3L1, *r* = − 0.45, *p* = 0.003 and Iba1, *r* = − 0.66, *p* = 0.003) (Fig. 6e, f) and perceptual speed (CHI3L1, *r* = − 0.46, *p* = 0.002 and Iba1, *r* = − 0.69, *p* = 0.002) (Fig. 6g, h) across groups.

## Discussion

Although studies implicate chitinase proteins and NPTX2 in the pathogenesis of AD, their role in preclinical AD remains under-investigated. In the present study, we found that the numbers of FC CHI3L1-positive astrocytes, mainly in gray matter layers I and II and WM, were unchanged in MCI and mild AD, whereas in sAD, CHI3L1 cell numbers were significantly increased in all cortical layers and WM. Furthermore, FC CHI3L1 protein levels showed a positive trend to increase during disease progression, whereas CHI3L2 was significantly reduced in sAD compared to NCI. In contrast, others have shown that protein and RNA levels for these chitinases were increased in the entorhinal cortex, hippocampus, superior frontal gyrus, primary visual cortex, middle temporal gyrus, and posterior cingulate cortex in advanced AD compared to age-matched controls [74]. These observations, when taken together, suggest that chitinase levels vary between cortical regions, perhaps related to the extent and type of AD pathology. Although several studies revealed an increase in CSF CHI3L1 levels, which correlated with CSF markers for tau and amyloid in AD [75, 76], we did not find a correlation between CHI3L1 levels/counts and neuritic and diffuse plaques or NFTs. Similar to that in other reports [77], we observed CHI3L1-positive astrocytes in close apposition to amyloid plaques in MCI and AD. However, many CHI3L1-ir astrocytes were independent of plaque pathology, suggesting that amyloid is not a necessary precondition for the onset of CHI3L1 expression in astrocytes. Moreover, we demonstrated that CHI3L1 was present in a subset of GFAP astrocytes, but neither CHI3L1 nor CHI3L2 protein levels were related to GFAP during the progression AD. In addition, GFAP levels correlated with the astrocytic surface glycoprotein adhesion molecule CD44 and the latter negatively correlated with CHI3L2. Both CHI3L2 and CD44 showed opposite effects that reached significant levels in later stages of AD.

Interestingly, cortical CD44 was reported in a subset of astrocytes in FC layers I–II and WM in humans without cognitive impairment [78], similar to the CHI3L1 distribution observed in the present NCI cases. Although the distributions of CD44-positive and CHI3L1-positive astrocytes were similar, these two astrocytic markers did not colocalize. A previous study reported that CD44-positive astrocytes were increased and associated with Aβ plaques in AD [79]. In the present study, although astrocytes surrounding plaques expressed GFAP, not all GFAP-positive astrocytes displayed CHI3L1 or CD44. Functionally, CD44 and chitinase-like proteins are implicated in nervous system development, homeostasis, repair, and response to injury [80, 81] and have the same natural ligand, hyaluronan, a component of the extracellular matrix [82]. Since we found a strong correlation between CHI3L2 and CD44 during disease progression, we speculate that the expression of these markers in astrocytes is linked to extracellular matrix disturbances. The exact mechanism that triggers the expression of these inflammatory markers and their relationship with amyloid in AD requires further investigation.

We found that NPTX2 correlated negatively with plaque load and was significantly higher in mAD cases with high Braak scores. Interestingly, toxic, soluble Aβ oligomers disrupt glutamatergic synaptic function that leads to cognitive deficits [83–85]. NPTX2 also occurs in cortical pyramidal neurons, which are severely affected by tau aggregation in AD [86, 87], suggesting a role for NPTX2 in NFT formation. These observations and the present findings of a negative correlation between NPTX2 levels and plaque load, suggests that Aβ deposition modifies NPTX2 production. The significant correlation between CHI3L1 and NPTX2 in MCI and NCI suggests an interaction between astrocytes and neurons during the early stage of cognitive decline. Interestingly, changes in CSF CHI3L1 and NPTX2 levels are potential biomarkers for AD [88, 89] and MCI [22].

Here, we report a correlation between CHI3L1 and C1q protein levels in early AD. C1q is the first component of the complement pathway [90], and microglia and astrocytes are sources of C1q in the AD brain [91, 92]. Complement-associated factors are implicated in pathogen presentation, neurodegeneration, and microglia resolution of tissue injury [93]. Here, we found a strong correlation between C1q and microglia Iba1 protein levels during AD progression. Microglia activation, associated with high Iba1 levels that modulate synapse loss [94] and microglia reactivity, maybe related to the induction of pro-inflammatory genes and the expression of complement-associated factors following neuronal death [95]. Perhaps, microglia activation together with C1q expression activates CHI3L1 in astrocytes, which in turn plays a role in neuronal repair during AD progression.

We found a significant increase in Iba1 and GFAP profile numbers in the WM in MCI compared to NCI. We also observed a strong negative correlation between CHI3L1-positive astrocytes and Iba1-positive microglia numbers and perceptual speed and episodic memory [96] in early AD, indicating an association between WM degeneration and early memory deficits in AD [97–100]. Since CHI3L1 is highly expressed in WM perivascular astrocytes [101] and is implicated in angiogenesis [102], it is possible that it plays a role in blood vessel conservation and remodeling during disease onset.

Furthermore, hypoperfusion induced by small-vessel disruption leads to degeneration of astrocytes and fibrosis of the extracellular matrix [103]. Since blood-brain barrier (BBB) dysfunction is related to WM damage [104] and CHI3L1 is related to BBB disruption [105], we suggest that increased CHI3L1 expression in perivascular astrocytes is an attempt to remodel the blood vasculature. Perhaps a breakdown of the BBB increases CSF CHI3L1 levels, which in turn is related to cognitive dysfunction in preclinical AD [106].

The present study found a relationship between CHI3L1 and NPTX2 levels in the onset AD, further supporting CSF levels of these proteins as biomarkers for AD [88, 89] and MCI [22]. NPTX2 levels marked MCI conversion to AD [107], improved diagnostic classification of AD, and predicted cognitive performance in MCI and AD [108]. Together, these observations suggest that CHI3L1 and NPTX2 should be considered as novel biomarkers to improve the diagnosis and prediction of cognitive decline during the progression of AD.

## Conclusion

In summary, we demonstrated that both FC CHI3L1-ir astrocytic number and CHI3L2 and CD44 protein levels were altered in sAD. However, white matter Iba1- and GFAP-ir cell numbers were increased in MCI. Additionally, white matter CHI3L1- and Iba1-ir glial cell numbers were associated with cognitive performance during disease progression. These results suggest that WM inflammation occurs earlier than in gray matter and CHI3L1 plays a critical role in WM neuroinflammation associated with cognitive decline in AD.

## Data Availability

The conclusions in the manuscript are supported by the data presented in the text and figures.

## Abbreviations

AD: Alzheimer’s disease
APP: Amyloid precursor protein
Aβ: Amyloid beta
C1q: Complement component 1q
CERAD: Consortium to Establish a Registry for Alzheimer’s Disease
CHI3L1 (or YKL-40 or HC gp-39): Chitinase 3-like 1
CHI3L2 (or YKL-39): Chitinase 3-like 2
CSF: Cerebrospinal fluid
FC: Frontal cortex
GFAP: Glial fibrillary acidic protein
Iba1: Ionized calcium-binding adapter molecule 1
-ir: -Immunoreactive
mAD: Mild to moderate Alzheimer’s disease
MCI: Mild cognitive impairment
NCI: Nocognitive impairment
NFT: Neurofibrillary tangle
NPTX2 (or Narp): Pentraxin II
RT: Room temperature
sAD: Severe Alzheimer’s disease
TREM2: Triggering receptor expressed on myeloid cells 2
WM: White matter
